# Quantitative comparison of fungal genome assembly strategies using short and long reads from simulated and empirical sequencing data

**DOI:** 10.1099/mgen.0.001824

**Published:** 2026-08-26

**Authors:** Gabriel Amorim de Albuquerque Silva, Temitope R. Folorunso, Lori G. Eckhardt, Janna R. Willoughby

**Affiliations:** 1 College of Forestry, Wildlife and Environment, Auburn University, Auburn, AL, USA

**Keywords:** benchmarking, fungal genomics, genome assembly, sequencing depth

## Abstract

High-quality fungal reference genomes are essential for comparative, functional and evolutionary studies, yet fungal genome features such as repeats, structural rearrangements, accessory chromosomes and intron-rich genes can complicate genome assembly and the selection of cost-effective sequencing strategies. Here, we benchmark fungal genome assembly performance using simulated and empirical short- and long-reads datasets to evaluate how sequencing depth, assembler choice and genome characteristics influence contiguity, completeness, accuracy and computational requirements. Using simulated reads from complete fungal genomes spanning diverse sizes and compositions, we evaluated short-reads (SR), long-reads (LR), hybrid and polished LR assemblies across sequencing depths from 10X to 100X. Key trends were validated using empirical sequencing data from 10 fungal isolates assembled with multiple strategies, including different Flye assembler parameter sensitivity and SR polishing. Across datasets, LR produced the largest improvements in contiguity, with most gains achieved at ~20–40X coverage and diminishing returns beyond moderate depth. SR polishing substantially improved base-level accuracy at relatively low cost, with ~10–20X coverage often sufficient to approach maximal error reduction. Hybrid assemblers showed strong algorithmic variability, with trade-offs between contiguity, error rates and computational demand. Genome architecture also influenced outcomes, as larger and more feature-dense genomes benefited more from long-read data while GC content had limited impact. Overall, our results suggest that moderate long-read coverage (~30–40X) combined with modest short-read polishing (~10–20X), particularly using Flye plus Polypolish, provides a strong balance of contiguity, completeness, accuracy and resource efficiency for generating high-quality fungal genome assemblies.

**Impact Statement**

Fungal genome sequencing is expanding rapidly across ecology, plant pathology, biotechnology and clinical and veterinary microbiology, yet experimental design decisions regarding sequencing depth, assembler selection and hybrid workflows are still largely guided by bacterial benchmarking studies or limited single-species comparisons. Because fungal genomes vary widely in size, repeat content and gene architecture, these assumptions can lead to inefficient sequencing strategies, increased computational costs and suboptimal assemblies. Here, we develop a reproducible assembly benchmarking framework that combines large-scale simulations from 66 complete fungal genomes spanning plant, animal and human-associated taxa with newly generated short- and long-read sequencing data from 10 field-collected isolates. This approach enables evaluation of assembler performance across diverse genome architectures and tests whether patterns identified in simulations translate to real biological datasets. Across both simulated and empirical datasets, we show that reliable fungal genome reconstruction can be achieved without excessive sequencing depth by identifying consistent performance thresholds. Assembly contiguity and completeness stabilize at moderate long-read coverage, after which improvements depend more strongly on assembler choice and genome structure than on additional data volume. Hybrid workflows show trade-offs in accuracy, contiguity and computational demand, whereas targeted short-read polishing provides an efficient strategy for improving base-level accuracy. These findings offer practical guidance for fungal genome assembly and support more robust downstream genomic analyses across non-model microbial systems, including ecologically, agriculturally and clinically important fungi.

## Data Summary

The reference fungal genomes used for simulation are available in the NCBI Assembly database under the accession numbers listed in Table S1. Empirical raw sequencing data generated for this study are deposited in the NCBI Sequence Read Archive (SRA) under accession PRJNA1474061.

All scripts used for read simulation, assembly, polishing, benchmarking and statistical analyses are available in the project GitHub repository (https://github.com/bielasilva/fungi_assembly_benchmarking). Software versions, parameters and workflow configurations are provided within the repository and detailed in the Methods.

## Introduction

Fungi are one of the most diverse and socioeconomically important groups of organisms, influencing biotechnology, agriculture, medicine and environmental science [1]. Their impacts span the production of pharmaceuticals such as antibiotics, statins and immunosuppressants [2, 3], industrial enzymes [4] and biocontrol agents [5], as well as negative impacts as pathogens that cause major losses in agriculture [6], timber industry [7] and livestock [8, 9], and pose health concerns for humans [10, 11], domestic animals [12] and natural forest ecosystems [13]. Given this ecological breadth and economic impact, elucidating how they respond to environmental stressors, interact with other species and evolve is fundamental to their sustainable use and management. High-quality reference genomes provide the foundation for these insights, enabling comparative, functional and evolutionary analyses across this extraordinarily diverse kingdom, and support clinical applications such as pathogen identification, antifungal resistance tracking and outbreak surveillance.

Despite their importance, high-quality reference genomes remain scarce for many fungal taxa, particularly among ecologically important plant pathogens, endophytes and soil-associated lineages where assemblies are often fragmented or unavailable [14, 15]. Although typically smaller than those of plants and animals, fungal genomes can greatly vary in size, organization and gene content [16]. Fungal genomes range from compact, haploid genomes of less than 10 Mb to large, to repetitive and polyploid genomes exceeding 1 Gb [15]. Examples at the small end include the human-associated pathogens *Malassezia restricta* [17] and *Pneumocystis jirovecii* [18], while large-genome species include the maize rust *Puccinia polysora* [19] and the cicada-infecting *Massospora cicadina* [20]. In addition, many species possess accessory or conditionally dispensable chromosomes that contribute to host specificity, virulence or environmental adaptation [21, 22]. Frequent structural rearrangements [23, 24], abundance of transposable elements [25], horizontal gene transfers [26] and complex gene clusters [27] create computational challenges to generating high-quality reference assemblies in these fungal species.

Because of the genomic complexity, the accuracy and completeness of fungal genome assemblies depend on the sequencing technology and assembly strategy. Traditionally, the choice of sequencing technology determines the assembly outcome and downstream analytical options. Illumina short-read (SR) sequencing has long been the gold standard for base-level accuracy (<0.1% error rates), but at the cost of limited resolution potential in repetitive regions, leading to fragmented assemblies [28]. In contrast, Oxford Nanopore Technologies long-reads (LRs) can exceed 100 kb, facilitating the resolution of complex repetitive elements and structural variants. Earlier nanopore chemistries had error rates exceeding 5–10%, which historically limited their use for high-accuracy applications [29], but successive improvements in pore chemistry, basecalling models and read length have substantially reduced raw-read error rates and yielded high consensus accuracy on bacterial genomes, comparable to or exceeding SR assemblies [30–32].

Given the historical limitations of single-platform assemblies, hybrid approaches have emerged as an alternative to the challenges of single technology assemblies. These methods combine the contiguity advantages of LRs with the base-level accuracy of SRs [33], and post-assembly polishing tools, such as Pilon [34] and Polypolish [35], using SRs can significantly improve the base-level accuracy of LR assemblies [36]. However, the optimal combination of assemblers, polishing tools and sequencing depths for fungal genomes remains poorly explored. Previous benchmarking studies have primarily focused on bacteria [36–39], with the few exceptions targeting single-taxon model eukaryotic systems (e.g. *Giardia* [40], *Saccharomyces* [41], *Drosophila* and human reads [42]) and limited subsets of assemblers and sequencing depths, leaving the distinctive challenges of fungal genome architecture largely unaddressed. This warrants a systematic, large-scale evaluation to guide cost-effective experimental design and accurate genome reconstruction.

This study addresses these critical knowledge gaps through a systematic comparison of fungal genome assembly approaches. First, we used simulated data of 66 fungal genomes to consider how sequencing strategy and coverage depth influence assembly size, contiguity, completeness, accuracy and computational cost in fungal genomes across four distinct sequencing and assembly strategies: SR-only, LR-only, hybrid and LR-polished, evaluating across multiple sequencing depths (10X to 100X coverage) to determine optimal resource allocation strategies. Next, we evaluated how intrinsic characteristics, including genome size, number of genes and coding elements, and GC content, modulated assembler performance. Trends observed within the simulated dataset were then empirically validaded using 10 different fungal isolates. Finally, we considered which combinations of sequencing technology, depth and assembly workflow supported the most efficient trade-offs between quality and computational resources to provide evidence-based recommendations for fungal genome assembly workflows. This was designed to enable researchers to make informed decisions about sequencing strategies and computational pipelines tailored to their research and resource availability.

## Methods

### Overview

We evaluated fungal genome assembly performance using a two-part framework that combined simulations with empirical validation. First, we measured assembly behaviour using simulated SR and LR sequencing data from complete fungal reference genomes and generated assemblies across multiple sequencing depths with representative SR-only, LR-only, hybrid and LR-plus-polishing strategies. These assemblies were compared using standardized metrics of contiguity, completeness, base-level accuracy, assembly size and computational resource requirements, and genome characteristics were incorporated to examine how intrinsic features influenced performance. Second, to evaluate how these patterns translated to empirical datasets, we sequenced 10 distinct fungal isolates with both SR and LR technologies and applied a focused subset of assembly workflows to assess depth-dependent trends. These isolates were treated as independent empirical genomes for benchmarking purposes, and species-level taxonomic assignments were not required for the assembly-performance comparisons and were not used as a factor in the analyses.

### Simulated genome data

We simulated SR and LR sequencing data from 66 complete fungal genomes, representing all available assemblies on NCBI RefSeq as of 29 July 2025 (Table S1, available in the online Supplementary Material). These reference genomes represented a range of taxa and genomic complexities, so we summarized key characteristics of the reference assemblies by quantifying GC content, total gene count, number of contigs and total assembly size. Assembly size, GC content and number of contigs were quantified by custom scripts and gene counts were predicted using GeneMark-ES [43]. Reads for each assembly were simulated using NanoSim v3.2.3 [44] for Nanopore and InSilicoSeq v2.0.1 [45] for Illumina (2×250 bp) at 100X coverage. Subsequently, seqtk v1.5 [46] was used to subsample the reads to 10X, 15X, 20X, 25X, 30X, 35X, 40X, 50X, 60X and 75X coverage.

Using the simulated reads, including downsampled datasets, we considered four complementary assembly strategies and compared performance among these approaches. We considered SR-only assemblers SPAdes v4.2.0 [47] and ABySS v2.3.10 [48] as well as LR-only assemblers Flye v2.9.6 [49], Canu v2.3 [50], Hifiasm v0.25.0 [51] and Raven v1.8.3 [52]. Following these, we considered hybrid assemblers ABySS Hybrid v2.3.10 [48], MaSuRCA v4.1.4 [53] and hybridSPAdes v4.2.0 [47]. Finally, we used the top-performing LR-only assembler to be SR polished by Pilon [34], Polypolish [35] or Racon [54]. Within this set, Racon was run with both bwa-mem2 v2.3 [55] or Minimap2 v2.30 [56] as mappers to assess the impact of the mapper on polishing. Each assembler or polisher was run closest to the recommended parameters and standardized computational resources (*-threads 10* or equivalent) to ensure comparability, and full scripts can be found on GitHub. Canu was also run with the *genomeSize* parameter set to the known genome size, as it is a required parameter.

### Empirical genome data

For the empirical data assessment, ten distinct fungal isolates were obtained from symptomatic pine needles collected from diseased trees in pine forests in the southeastern USA. These isolates were selected to provide independent empirical genome assemblies for workflow validation, as species-level taxonomic assignments were not used in the benchmarking analyses, and we therefore refer to them here as fungal isolates rather than as species. Sampling, isolation and culturing followed established protocols described in Folorunso *et al*. [57]. In short, infected needles were surface-sterilized, sectioned and plated on Malt Extract Agar medium to obtain pure fungal cultures. The isolates were cultured in liquid yeast extract peptone dextrose medium, the mycelia were collected and the DNA was extracted using a modified cetyltrimethylammonium bromide protocol optimized for fungal tissues rich in secondary metabolites and polysaccharides. The full step-by-step protocol is available in Folorunso *et al*. [57] and archived on Protocols.io [58].

Following the simulated data approach, we generated SR and LR sequencing data for each of the ten fungal isolates. For SRs, sequencing was carried out as 150 bp paired-end on Element Biosciences AVITI24 by the University of Minnesota Genomics Center. Raw SRs were processed using Fastp v0.24.1 [59] (*--correction --detect_adapter_for_pe --trim_front1 20 --trim_front2 20 --trim_tail1 2 --trim_tail2 2*). For LRs, we used the Oxford Nanopore Technologies MinION Mk1C or Mk1D, the Native Ligation Barcoding Kit 24 (SQK-NBD114.24) on flow cells R10.4.1 (FLO-MIN114). We basecalled with Dorado v0.9.1 (super-accuracy model v5.0.0) and filtered LRs with Chopper v0.11.0 [60]. No fixed number of bases were removed from the ends of reads, and filtering was limited to removing reads shorter than 1,000 bp (*--minlength 1000*). Based on simulated data output, we assembled using ABySS, SPAdes (SR-only and hybrid), Flye (with *--min-overlap* set to 1,000, 1,500, 2,000, 2,500 or 3,000) and Flye+Polypolish.

### Assembly comparisons

For simulated datasets, assembly performance was evaluated using reference-based metrics. Total assembly length, N50, number of contigs and base-level accuracy were quantified with QUAST v5.3.0 [61] and assembly completeness was assessed with BUSCO v6.0.0 [62] using the fungi_odb10 lineage dataset. Relative assembly size was calculated as the difference between the assembled genome length and the corresponding reference genome used to generate simulated reads, enabling standardized comparisons across assemblers and sequencing depths. Similarly, contiguity differences were assessed by comparing the number of contigs produced by each assembly to those of the reference genome. Because the true genome sequence was known, BUSCO completeness, genome fraction recovered, length error and base-level error rates were interpreted as direct measures of assembly accuracy relative to the reference.

For empirical datasets, where a ground-truth genome was unavailable, assemblies were compared using reference-free assembly metrics, k-mer-based genome size estimates and BUSCO gene completeness. Genome size was estimated independently from both SR and LR datasets using KMC v3.2.4 [63], followed by GenomeScope2 v2.0.1 [64]. Total assembly length was then compared across assemblers and sequencing depths and evaluated against these size estimates to assess concordance with expected genome size. N50 and contig counts were used to assess contiguity, while BUSCO completeness and multi-copy BUSCO percentages were used as proxies for gene recovery and potential duplication artefacts. Base-level accuracy metrics (mismatches and indels per 100 kbp) were primarily interpreted for the polishing comparison, where differences relative to the unpolished Flye assembly reflected the sequence changes introduced by polishing.

Finally, computational requirements (runtime and maximum random-access memory (RAM) usage) for each assembly approach were quantified by accessing SLURM accounting records (command *sacct*). For polishing strategies, runtime was calculated as the sum of the original assembler and the polisher (e.g. Flye=5 min, Racon=2 min and Flye+Racon=7 min), and maximum RAM was taken as the highest value across the runs (e.g. Flye=10 GB, Pilon=5 GB and Flye+Pilon=10 GB).

We summarized read and assembly metrics across fungal genomes using means and their associated 95% confidence intervals (CIs), which were used to draw the plots and are reported as mean±95% CI.

It is worth noting that two assemblers contributed fewer observations to the computational analyses due to practical execution limits. MaSuRCA failed to complete several assemblies with segmentation faults, an issue that has been reported previously [65], and appears to be dataset- and parameter-dependent. Canu, on the other hand, took over a month to assemble the largest sample at depths higher than 15X, exceeding the available computing infrastructure. Therefore, failed MaSuRCA and incomplete Canu executions were excluded from the analysis.

### Statistical analysis

Statistical analysis was conducted in R v4.4.1 [66] supplemented with tidyverse v2.0 [67] and janitor v2.2.1 [68]. As part of the initial exploratory analysis, we assessed correlations among the response variables using the pairwise Spearman test of the stats::cor() v3.6.2 package on the simulated single-strategy data. Next, assembly performance was analysed using linear mixed-effects models implemented in lme4 v1.1.37 [69]. Two model classes were fit, distinguished by the depth structure of the assembly strategy. Single-strategy models were used for SR-only assemblers (SPAdes, ABySS) and LR-only assemblers (Flye, Hifiasm, Canu, Raven, NextDenovo and Miniasm), with depth modelled as a continuous predictor (*depth_eff*) representing whichever sequencing technology was used. Multi-strategy models were used for hybrid assemblers (SPAdes Hybrid, MaSuRCA) and polishing combinations (e.g. Flye+Polypolish), with separate continuous predictors for LR depth (*depth_np*) and SR depth (*depth_il*) and their interaction.

Fixed effects in the single-strategy models included the assembler programme, sequencing depth, their interaction and a genome-characteristic covariate. For simulated data, additional genome features (size, GC content, gene density and coding sequence per gene [CDS-per-gene] ratio) and their interactions with programme and depth were included to evaluate how genome architecture modulated assembler performance. For empirical data, no reference genome was available, so the genome-size covariate was computed as the per-isolate mean assembly length across the five Flye assemblies (minimum-overlap settings 1,000–3,000 bp) at full sequencing depth. This estimate was applied for each isolate as a per-sample constant, and the corresponding full-depth Flye assemblies were excluded from the modelling dataset to avoid using the same observations for both response and covariate estimation. All continuous covariates were centred and scaled prior to fitting, and sample identity was included as a random intercept.

Response variables included contiguity (N50), completeness (BUSCO complete percentage, BUSCO difference from reference), genome recovery (genome fraction recovered, length error percentage), base-level accuracy (mismatches and indels per 100 kbp) and computational requirements (maximum RAM, runtime). Most responses were transformed prior to modelling to satisfy Gaussian residual assumptions: log10 for responses spanning multiple orders of magnitude (N50, runtime, RAM), logit (with a 0.01 shrinkage offset) for bounded percentage responses near the upper limit (BUSCO complete, genome fraction recovered) and log1p for non-negative rate counts (mismatches and indels per 100 kbp). Signed differences (BUSCO difference, length error percentage) were modelled on the original scale.

Significance of fixed effects and interactions was assessed using type III Wald *χ*² tests with car::Anova v3.1–5 [70]. Post hoc pairwise comparisons among assemblers and sequencing depths were performed with Tukey adjustments using emmeans v1.11.2.8 [71] and multcomp v1.4.28 [72]. Per-programme depth slopes and per-programme responses to genome covariates were extracted using emtrends. Residual diagnostics were assessed using simulated quantile residuals (*n*=1,000) with DHARMa v0.4.7

This allowed us to test whether assembly quality metrics responded differently to increasing sequencing depth across assemblers and whether genome characteristics influenced assembly outcomes. Additionally, we assessed residual diagnostics using simulated quantile residuals (*n*=1,000) with R package DHARMa v0.4.7 [73], which led to sensitivity analyses in the single-strategy model by refitting after excluding Miniasm (which produced extreme outlier values for several quality responses) and refitting separately within each strategy (SR and LR). To enable a direct comparison between the empirical and simulated datasets, we predicted values back-transformed to the original response scale. For the simulated dataset, we also derived thresholds from the models to better guide decision process on choosing the depth. Predictions were computed on the transformed response scale, at the mean of all genome-architecture covariates, and at depths from 10 to 100X in 5X intervals via lme4::bootMer with 1,000 bootstrap replicates to obtain 95% CIs. Thresholds for the decision guide were defined as the minimum depth at which 90% of the programme’s best predicted best value was achieved.

All analyses were run on the Auburn University’s Easley High Performance Computing Cluster using SLURM for job scheduling and resource management. All programs were run in nodes using Intel Xeon Gold 6248R processors with 48 cores and 192 GB of RAM. Jobs were submitted using the *sbatch* command, requesting 10 cores and 180 GB of RAM for each assembly. Assemblers were installed using Conda into separate environments, except for ABySS [48], which had its dependencies installed via Conda, and then manually compiled into the environment. Details on the installation and commands used for each programme can be found in the GitHub repository.

## Results

### Statistical analyses

Pairwise Spearman correlations among the response variables confirmed that most captured meaningfully distinct components of assembly quality (|*ρ*| < 0.5, Fig. S1). The principal exceptions were genome fraction recovered and length error percentage (*ρ*=0.86), which both quantify deviation from the reference genome length, and mismatches and indels per 100 kbp (*ρ*=0.79), which both measure base-level errors against the reference.

Initial single-strategy model adequacy assessed using DHARMa simulated residual diagnostics showed dispersion ratios within the expected range (0.95–1.00) across all models, indicating no over- or under-dispersion (Table S2). Kolmogorov–Smirnov tests of residual uniformity were statistically significant for all models, as expected given the large per-model sample sizes (*n*>5,000), but effect sizes were modest for most responses (KS statistic 0.05–0.18), indicating virtually acceptable fit. BUSCO completeness difference showed the largest deviation (KS=0.29), due to its strongly skewed distribution in which most assemblies achieved high recovery, even at lower depths.

To confirm that these residual deviations did not affect our conclusions, we refitted the models within each sequencing strategy, where the response distributions were more homogeneous. Similar fixed effects remained significant across all model variants (Table S3), and within-strategy models for genome fraction, length error percentage and mismatches per 100 kbp showed substantially improved residual uniformity (Table S2), confirming that conclusions about assembler-specific performance were robust to residual deviations.

### Simulated data outcomes

The 66 reference fungal genomes we identified via NCBI included a wide array of structural and compositional characteristics (Fig. S2). Genome length varied widely (7.4–122.8 Mbp) and was strongly associated with predicted gene content (*R*^2^=0.92, Fig. S2A). Although gene content and genome size were strongly associated, this relationship was not completely reflected in the number of coding sequences and introns, as above ~20 Mbp, the number of predicted CDS and intron features increased more rapidly than the number of genes. This likely reflects the higher compactness of smaller genomes, whereas larger genomes tend to accommodate longer genes, higher intron density and more complex exon-intron architectures, allowing individual genes to have multiple coding regions [16]. Furthermore, the references ranged from 3 to 23 contigs (average 9.6, Fig. S2B), and GC content showed substantial variation among samples, ranging from 28.3 to 62.3% (average 46.7%, Fig. S2C).

**Fig. 1.**
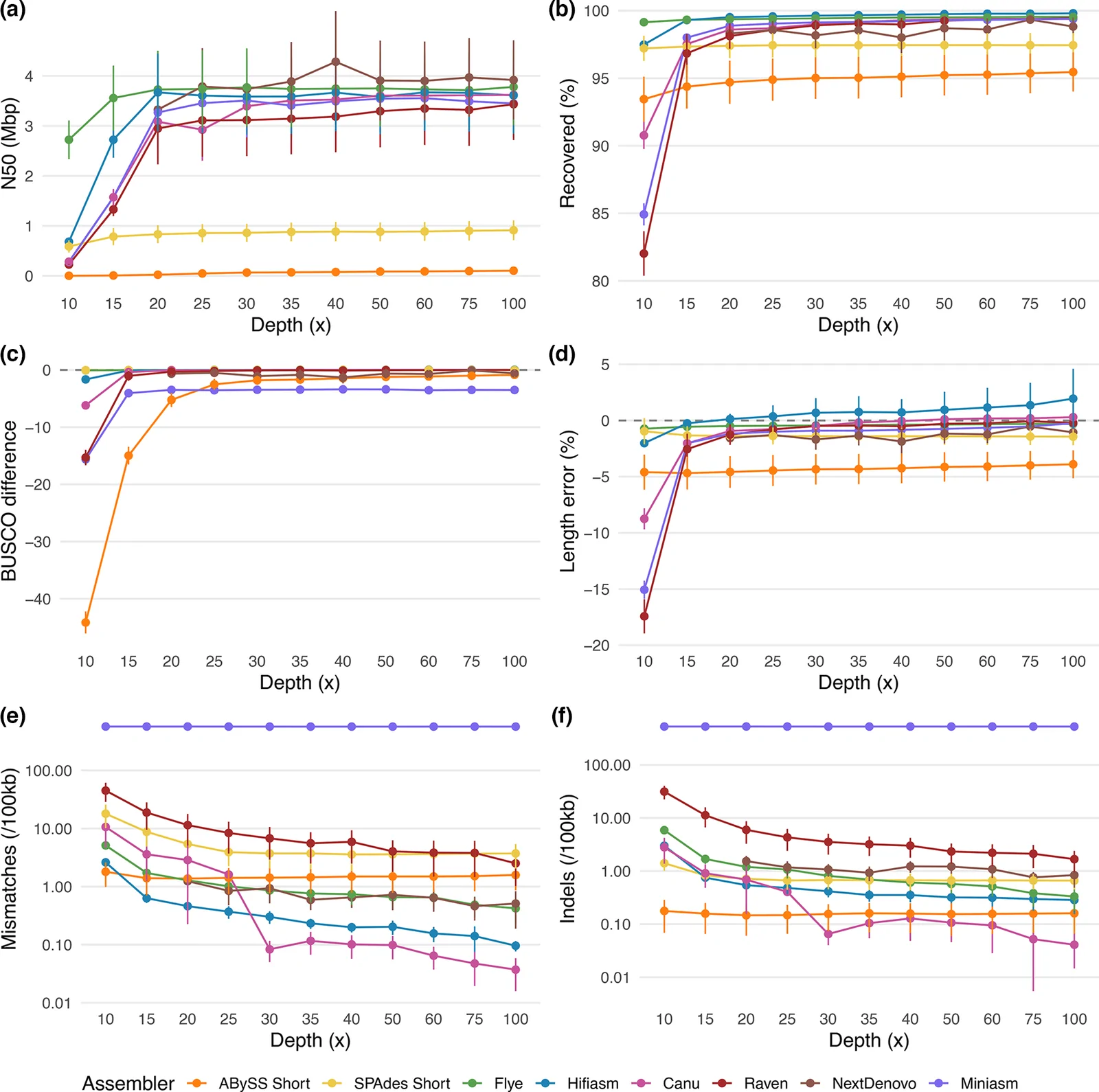
Assembly quality metrics for single assembly strategies. Panels show (**a**) N50, (**b**) genome fraction recovered, (**c**) BUSCO difference, (**d**) length error, (**e**) mismatches per 100 kbp and (**f**) indels per 100 kbp for each assembler as a function of sequencing depth. Error bars represent 95% CIs around the mean. Note the y-axis on panels (e) and (f) have been log scaled for better visualization. Overall, LR assemblers showed rapid improvements in contiguity, completeness and accuracy up to ~20–40× depth, after which gains plateaued, whereas SR assemblers exhibited minimal depth-dependent improvement.

Across single-technology assemblers, contiguity differed sharply by sequencing technology, with LR assemblers showing steep N50 gains between 10X and ~20–30X LR depth before plateauing, whereas SR assemblers showed minimal response to increasing depth (Fig. 1a). For example, Flye increased from 2.72 Mbp (±0.39) at 10X to 3.73 Mbp (±0.77) at 20X and remained stable through 100X. Canu showed an even sharper early rise from 10X to 20X (0.28 Mbp ±0.14 to 3.08 Mbp ±0.65), while SPAdes improved only modestly across depths (0.59 Mbp ±0.12 at 10X to 0.91 Mbp ±0.20 at 100X) and ABySS remained highly fragmented (<0.11 Mbp at all depths). This difference was confirmed by a strong interaction between assembler and sequencing depth (*P*<0.001, Fig. S3A), consistent with LR assemblers using read length to establish contiguity rapidly at moderate depth, while SR assemblers remain limited by repeat-spanning regardless of coverage. From a practical standpoint, LR contiguity is largely established by 20–40X, and the model-based threshold analysis (Table 1) confirms that Flye and NextDenovo reach 90% of their predicted best N50 by 20X and 10X depth respectively, with further investment offering diminishing returns.

**Table 1.** Model-based sequencing depth thresholds for each combination of assembler and response variable

Metric	N50	BUSCO completeness	BUSCO difference	Genome recovery	Length error	Mismatch	Indel
**ABySS short**	100	10	70	10	60	10	10
**Canu**	95	10	65	10	65	75	80
**Flye**	20	10	55	10	100	100	95
**Hifiasm**	85	10	60	10	25	85	100
**Miniasm**	95	10	100	10	70	10	10
**NextDenovo**	10	10	95	10	95	95	90
**Raven**	95	10	65	10	65	100	100
**SPAdes short**	65	10	75	10	10	95	85
**ABySS hybrid**	10 | 100	10 | 10	10 | 70	10 | 10	10 | 65	10 | 10	10 | 10
**Flye+Pilon**	20 | 10	10 | 10	45 | 45	10 | 10	95 | 45	100 | 80	100 | 75
**Flye+Polypolish**	20 | 10	10 | 10	45 | 80	10 | 10	95 | 30	100 | 10	100 | 10
**Flye+Racon** (**BWA**)	20 | 10	10 | 10	55 | 50	10 | 10	95 | 15	100 | 85	100 | 10
**Flye+Racon** (**Minimap2**)	20 | 10	10 | 10	45 | 80	10 | 10	95 | 10	95 | 100	85 | 95
**MaSuRCA**	65 | 100	10 | 10	85 | 15	10 | 10	95 | 10	10 | 100	10 | 100
**SPAdes hybrid**	10 | 75	10 | 10	100 | 95	10 | 10	10 | 10	10 | 95	10 | 85

Each cell reports the lowest sequencing depth (in X coverage) at which the mixed-effects model first predicts the response to reach within 90% of its best value. Single-strategy programmes are reported with their only corresponding value, while multi-strategy programmes are reported as ‘LR | SR’. Threshold values should be interpreted along with the response values, as low thresholds combined with poor metrics potentially indicate programme limitations rather than efficient saturation.

On the other hand, completeness metrics saturated across all assemblers once moderate depth was reached. By 50X, Flye recovered 99.50% (±0.27), Hifiasm 99.75% (±0.17), Raven 99.26% (±0.29) and Canu 99.35% (±0.23) of the reference genome, with differences typically <0.5% points. In contrast, SR assemblers remained slightly lower even at high depth (SPAdes 97.45%±0.89; ABySS 95.46%±1.44 at 100X) (Fig. 1b). BUSCO differences relative to the reference similarly stabilized beyond ~20X for most LR assemblers (within ±0.1 pp), whereas Miniasm often underperformed (−15.6 pp ±0.93 at 10X and still reduced at high depth) (Fig. 1c). Significant ‘assembler×depth’ interaction for BUSCO completeness and genome recovery were found (*P*<0.001, Fig. S3A), and the threshold analysis confirmed 10X depth to satisfy every assembler tested. These results indicate that sufficient depth is required to approach full completeness, but algorithmic differences become the primary source of variation once moderate coverage is achieved.

Furthermore, base-level accuracy per 100 kbp (Fig. 1e, f) varied widely by the assembler. At 50X, Flye averaged 0.66 mismatches (±0.23) and 0.57 indels (±0.19) per 100 kbp, whereas Canu achieved even lower values (0.10±0.04 mismatches; 0.11±0.06 indels) (Fig. 1e). Hifiasm similarly showed low mismatch rates (<0.2 per 100 kbp at ≥40X) but exhibited slight genome length inflation at high coverage (+1.95%±2.66 at 100X) (Fig. 1d). Miniasm stood out by retaining extremely high error rates (>500 mismatches and indels per 100 kbp at 100X) (Fig. 1e, f), explaining its poor BUSCO performance despite moderate contiguity. This stems from the algorithm producing unpolished and uncorrected contig sequences from raw read, in an attempt to reduce computational load [74]. Contrary to expectations, SR assemblers did not achieve the lowest error rates: at 100X, SPAdes had 3.73 mismatches and 0.66 indels, while ABySS had 1.59 mismatches and 0.16 indels. They also consistently remained under-sized (SPAdes −1.44%±1.14; ABySS −3.89%±0.76 at 100X), likely due to collapsing of repeating regions. Assembler choice explained most variation in mismatches and indels, with secondary contributions from depth and their interactions (all *P*<0.001, Fig. S3A; per-programme slopes in Fig. S4). This suggests that LR assemblers can achieve comparable or better base accuracy than SR methods once sufficient depth is reached, while also better preserving the assembly length.

**Fig. 2.**
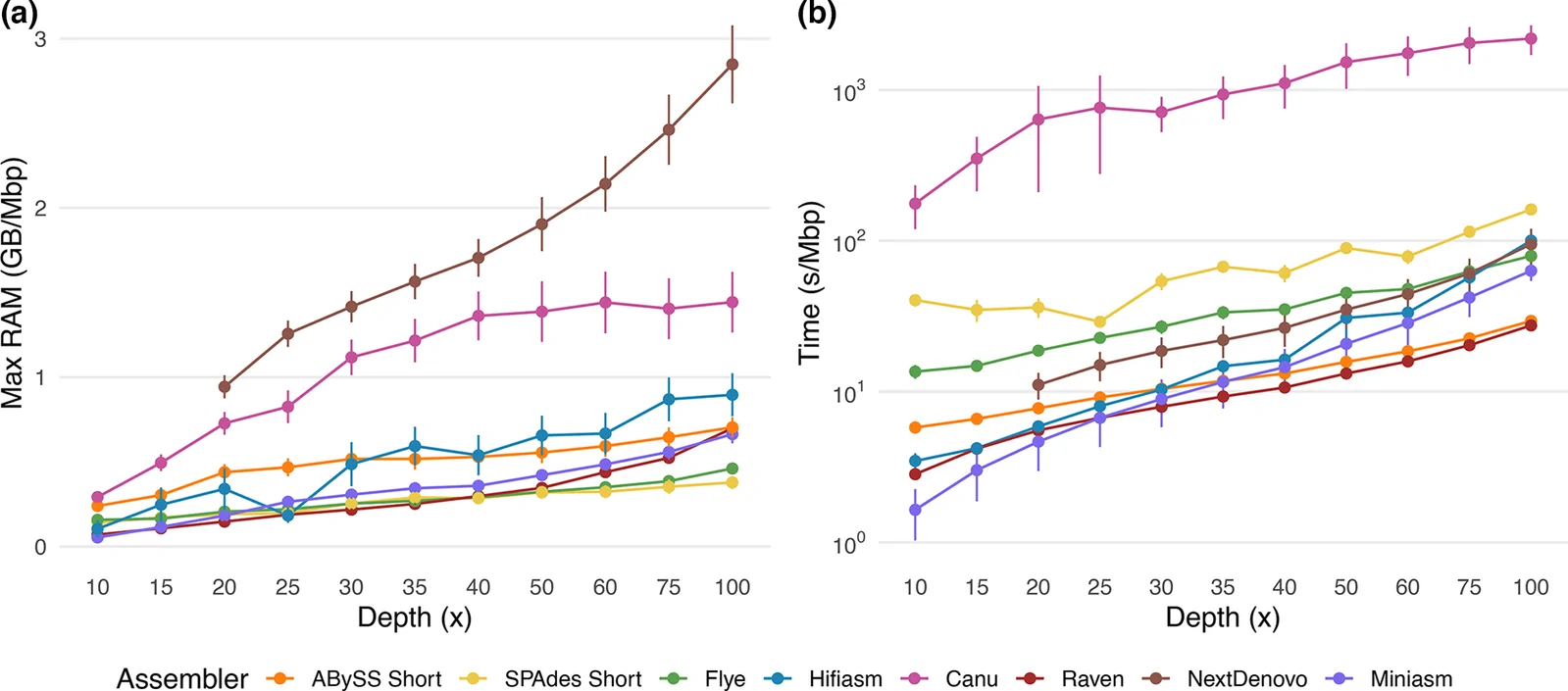
Normalized resource usage per Mbp of assembly for single assembly strategies. Panels show (**a**) maximum RAM usage per Mbp of assembly and (**b**) run time per Mbp of assembly for each assembler as a function of sequencing depth. Error bars represent 95% CIs around the mean. Note the y-axis on panel (b) has been log scaled for better visualization. Computational demand increased with sequencing depth but varied strongly by assembler, with Canu and Hifiasm requiring substantially more memory and runtime than other LR assemblers, while SPAdes remained comparatively slow despite low memory usage.

Computational performance varied substantially among assemblers, as maximum RAM and run time grew with depth and reflected differences in the algorithms rather than sequencing technology (Fig. 2). For normalized runtime (time per Mbp), the mixed-effects model detected a highly significant ‘assembler×depth’ interaction (*P*<0.001), confirming that scaling with depth differed among assemblers (Fig. S3A). Additionally, normalizing by genome size drastically reduced the within-assembler variance in runtime, since larger reference genomes incurred proportionally longer runtimes, as confirmed by the significance of the reference size alone in the mixed model for raw time (*P*<0.001), reinforcing that genome size is a major driver in resource usage. Normalized memory usage showed strong ‘assembler×depth’ effects (*P*<0.001) (Figs 2a and S2A), as at 50X, Flye used 0.32 GB/Mbp (±0.02), Hifiasm 0.66 GB/Mbp (±0.12), Raven 0.35 GB/Mbp (±0.01) and Canu 1.39 GB/Mbp (±0.18). SR assemblers were relatively memory-efficient (SPAdes 0.38 GB/Mbp ±0.04 at 100X) but comparatively slow at high coverage (161.2 s/Mbp ±10.8). Canu showed notably higher RAM requirements and longer runtimes, the latter sufficiently extreme to require separate scaling. At 50X, Flye required 45.1 s/Mbp (±4.06), Hifiasm 30.7 s/Mbp (±8.46) and Raven 13.2 s/Mbp (±0.47), whereas Canu required 1,527 s/Mbp (±512) (Fig. 2b). Together, these results indicate that while sequencing depth drives improvements in contiguity and completeness up to moderate coverage, computational burden and final assembly quality are largely determined by algorithmic design rather than sequencing technology alone.

**Fig. 3.**
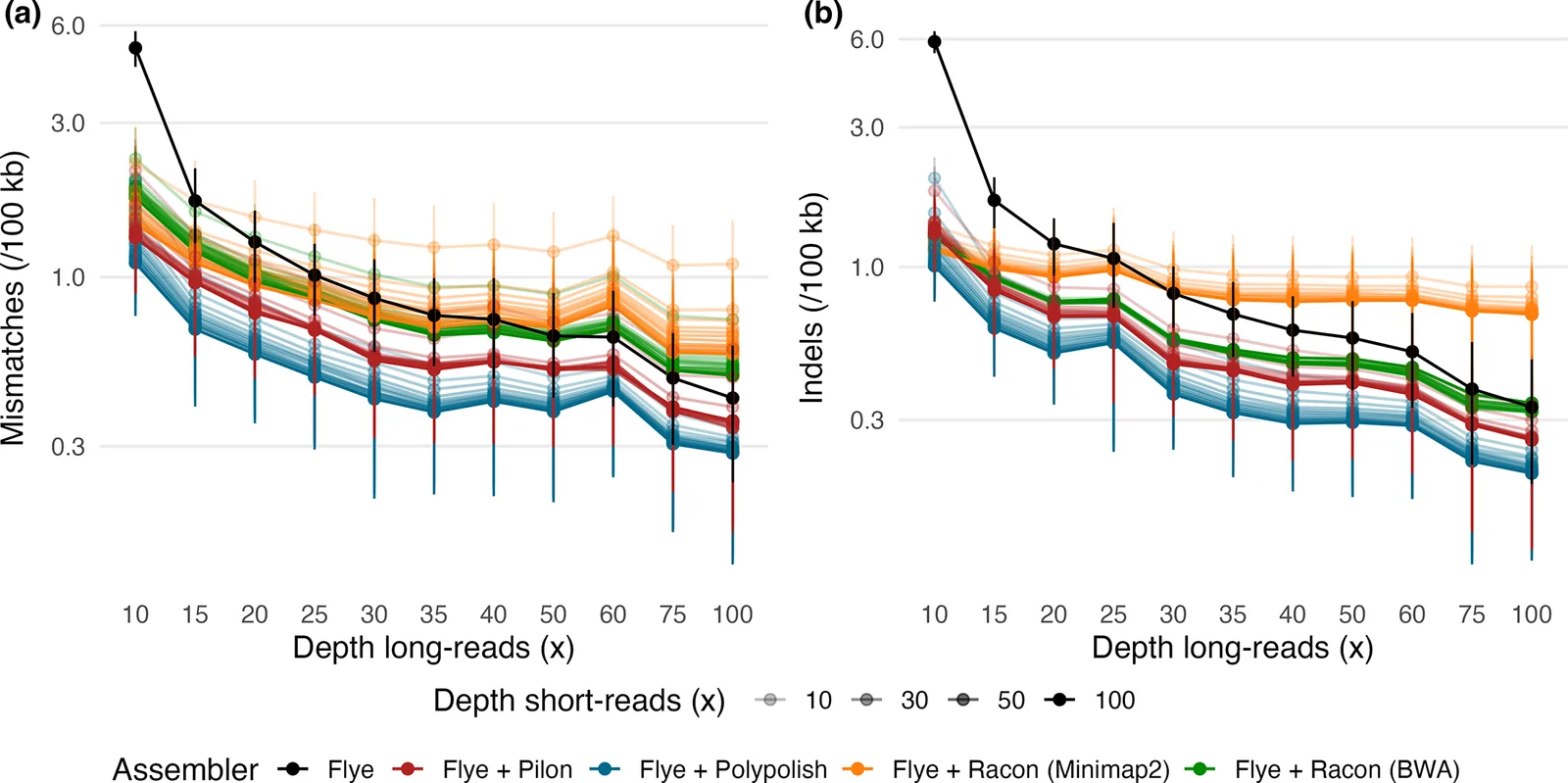
Assembly quality metrics for Flye assemblies before and after SR polishing. Panels show (**a**) mismatches per 100 kbp of the original and (**b**) indels per 100 kbp relative to the original reference genome for the unpolished Flye assembly and Flye assemblies polished with Pilon, Polypolish or Racon. Racon was evaluated using either Minimap2 or BWA-MEM2 for SR alignment. Error bars represent 95% CIs around the mean, x-axis indicates LR depth and transparency indicates SR depth. Note the y-axis on all panels has been log scaled for better visualization. Polishing noticeably reduced base-level errors, especially at low LR coverage, with most improvements at moderate SR depth and lesser gains beyond ~20–30X.

Due to Flye’s overall better performance as a stand-alone assembler, we selected it as a representative LR assembler and added a polishing step with SRs. This strategy clearly reduced small-variant errors while preserving contiguity (Fig. 3). Contiguity (N50) was primarily driven by the LR depth (*P*<0.001), with no detectable effect of SR depth or polishing programme (Fig. S5a, b), and genome recovery followed the same pattern. These observations are consistent with the purpose of the polishers to leverage the SRs to correct at nucleotide-level errors.

Polishing dramatically reduced base-level errors, particularly when applied to lower-quality LR assemblies. SR depth, LR depth and polishing programme effects were all highly significant (*P*<0.001, Fig. S5A, B), while genome architecture covariates had minimal influence on error rates (Fig. S5C–F). Using Pilon with 10X SR on the 10X LR unpolished Flye reduced errors to 2.13 mismatches and 1.82 indels per 100 kbp (±0.53 and ±0.38), whereas Polypolish achieved 1.99 and 2.01 (±0.35 and ±0.28) at the same depths. Increasing SRs depth to 60X further reduced errors, particularly at low LR depth. For example, Polypolish improved from 1.99 mismatches at 10X SR to 1.18 at 60X SR, on the same 10X LR Flye assembly, while at 40X LR+60X SR reached ~0.55 mismatches and 0.40 indels per 100 kbp. However, the magnitude of improvement diminished at higher LR depths, indicating that polishing primarily compensates for limited LR accuracy rather than enhancing already high-quality assemblies. Accordingly, strong base-level accuracy could be achieved at low sequencing cost, provided that Nanopore depth is sufficient to establish contiguity. The model-based threshold analysis (Table 1) also highlighted an eightfold difference between Polypolish and Pilon in SR depth requirement in mismatch reduction (10X versus 80X SR, respectively), suggesting that Polypolish is more efficient in extracting the correction from SRs than Pilon.

Additionally, racon-based polishing showed variable outcomes, as its choice of mapper had a significant impact (Figs 3 and S5). BWA-MEM2 outperformed BWA-MEM2 in reducing both mismatches (mean difference=0.065, *P*<0.001) and indels (mean difference=0.27, *P*<0.001). Notably, Racon (Minimap2) was the only combination influenced by genome size (*P*<0.05, Fig. S5C), evidencing that mapping algorithms differ on how they handle the data. This further demonstrates that each step in the pipeline can significantly influence the final result, even choices that might appear trivial, such as mapper selection.

Since polishing targets corrections at base-level, SR impacts on additional quality metrics (N50, length error and genome recovery fraction) were non-significant (Figs S3B and S6) and only marginally altered BUSCO differences. This pattern is consistent with indel correction at lower Nanopore depth improving gene-length integrity and downstream BUSCO alignments.

**Fig. 4.**
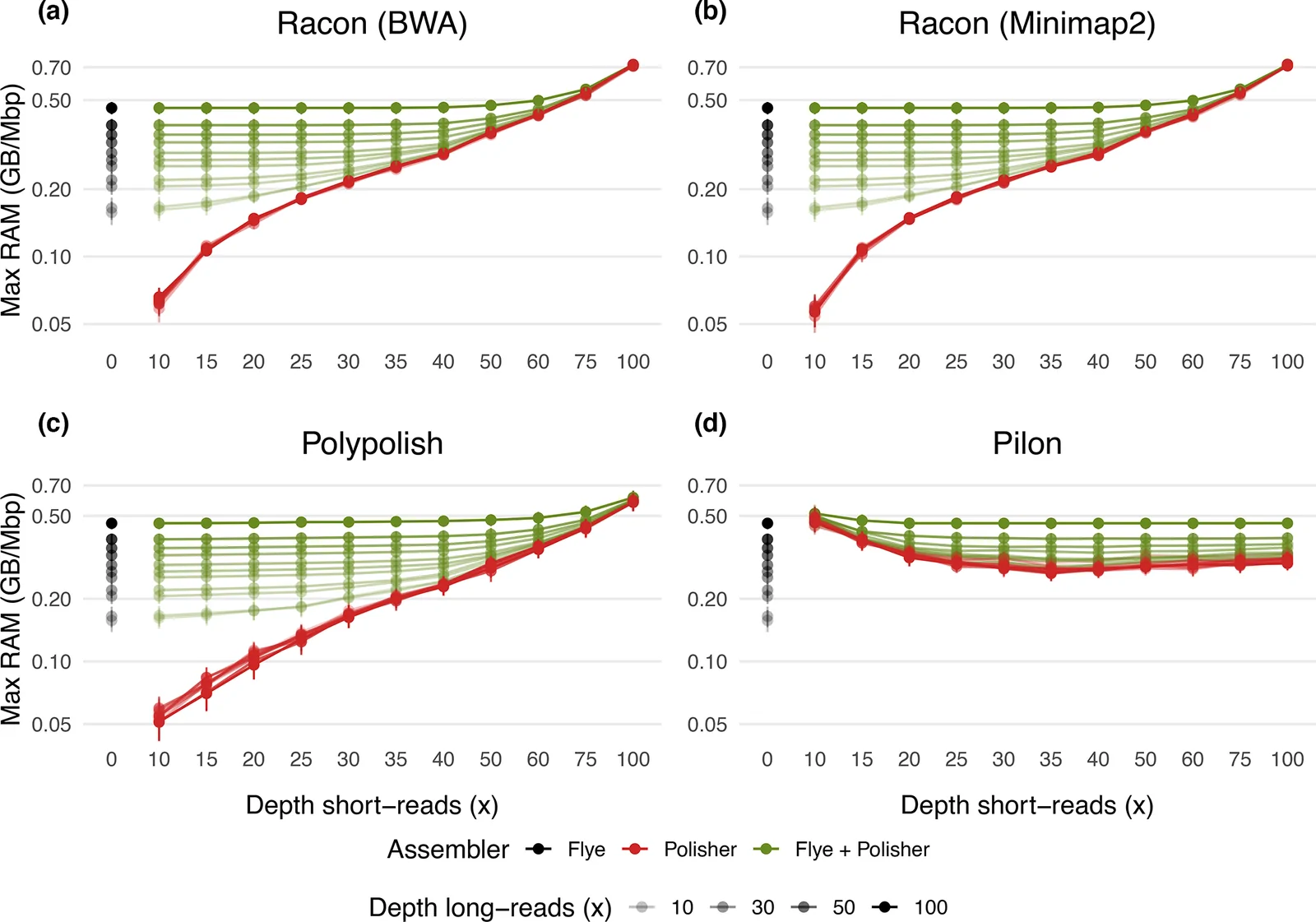
Normalized maximum RAM usage per Mbp for polished assembly strategies. Panels show (**a**) Racon with BWA-MEM2, (**b**) Racon with Minimap2, (**c**) Polypolish and (**d**) Pilon. Error bars represent 95% CIs around the mean, x-axis indicates SR depth and transparency indicates LR depth. Note the y-axis on all panels has been log scaled for better visualization. RAM usage generally increased with SR depth for Racon and Polypolish, whereas Pilon showed an inverse pattern with higher memory demand at low coverage, highlighting tool-specific behaviours.

**Fig. 5.**
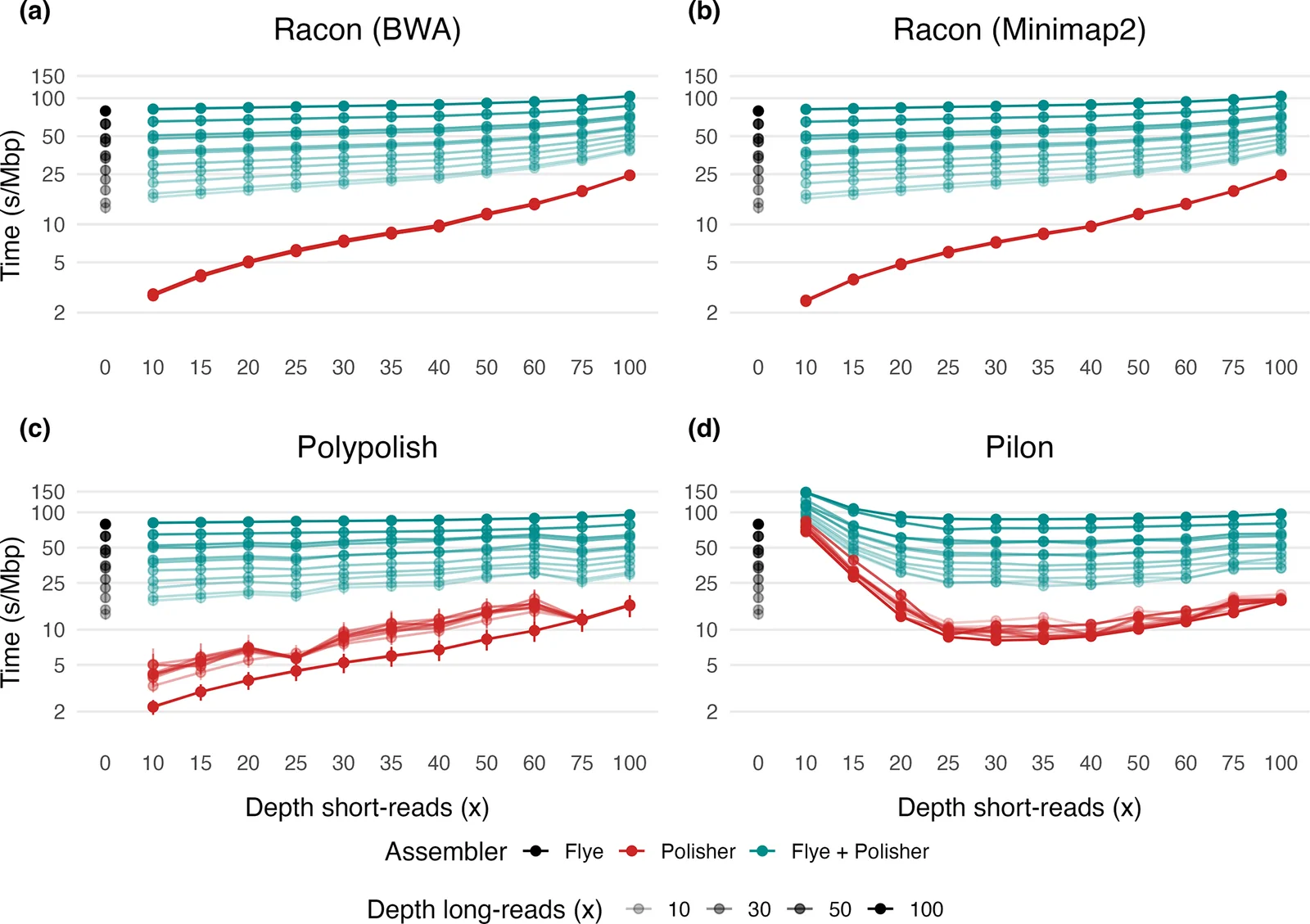
Normalized run time per Mbp for polished assembly strategies. Panels show (**a**) Racon with BWA-MEM2, (**b**) Racon with Minimap2, (**c**) Polypolish and (**d**) Pilon. Error bars represent 95% CIs around the mean, x-axis indicates SR depth and transparency indicates LR depth. Note the y-axis on both panels have been log scaled for better visualization. Runtime generally increased with SR depth for Racon and Polypolish, whereas Pilon showed the opposite pattern, with longer runtimes at low coverage that declined and stabilized as coverage increased.

Polishing added modest computational overhead to the underlying Flye assembly, with both RAM and runtime scaling primarily with SR depth (Figs. 4 and 5). For Racon-based approaches and Polypolish, memory scaled predictably with SR depth from ~0.05 GB/Mbp at 10X SR to 0.35–0.42 GB/Mbp at 60X SR (Fig. 4a–c), reinforcing that read alignment burden increases with SR depth. Runtime followed similar patterns, with each polisher adding 11–15 s/Mbp at 60X SR. All resource effects were significant for both polishing programme and depth (all *P*<0.001, Figs S3B and S5A, B) but dominated by the underlying LR assembler at low SR coverage and by the polishing step itself when SR depth exceeded LR depth (Figs. 4 and 5).

Unexpectedly, Pilon displayed an opposite pattern (Fig. 4d), where RAM usage was higher at low Illumina depth [0.51 (±0.06) GB/Mbp at 10X], then decreased with increasing depth, stabilizing beyond ~30X [0.33 (±0.03) GB/Mbp]. This inverse relationship contrasts with the other polishers and suggests that Pilon’s algorithm may operate less efficiently when coverage is low, possibly due to repeated re-evaluation of ambiguous regions with low support. Consequently, Pilon often required more memory than Flye itself at low SR depth. Considering these outcomes, polishing with SR generally added modest computational overhead to LR assemblies, with RAM and runtime scaling primarily with SR depth, except for Pilon which shows inverse trends likely due to its algorithmic design. Polypolish appeared to offer the best accuracy improvements with moderate resource demands, thus Polypolish-polished Flye assemblies were used as the benchmark LR baseline for subsequent hybrid comparisons.

**Fig. 6.**
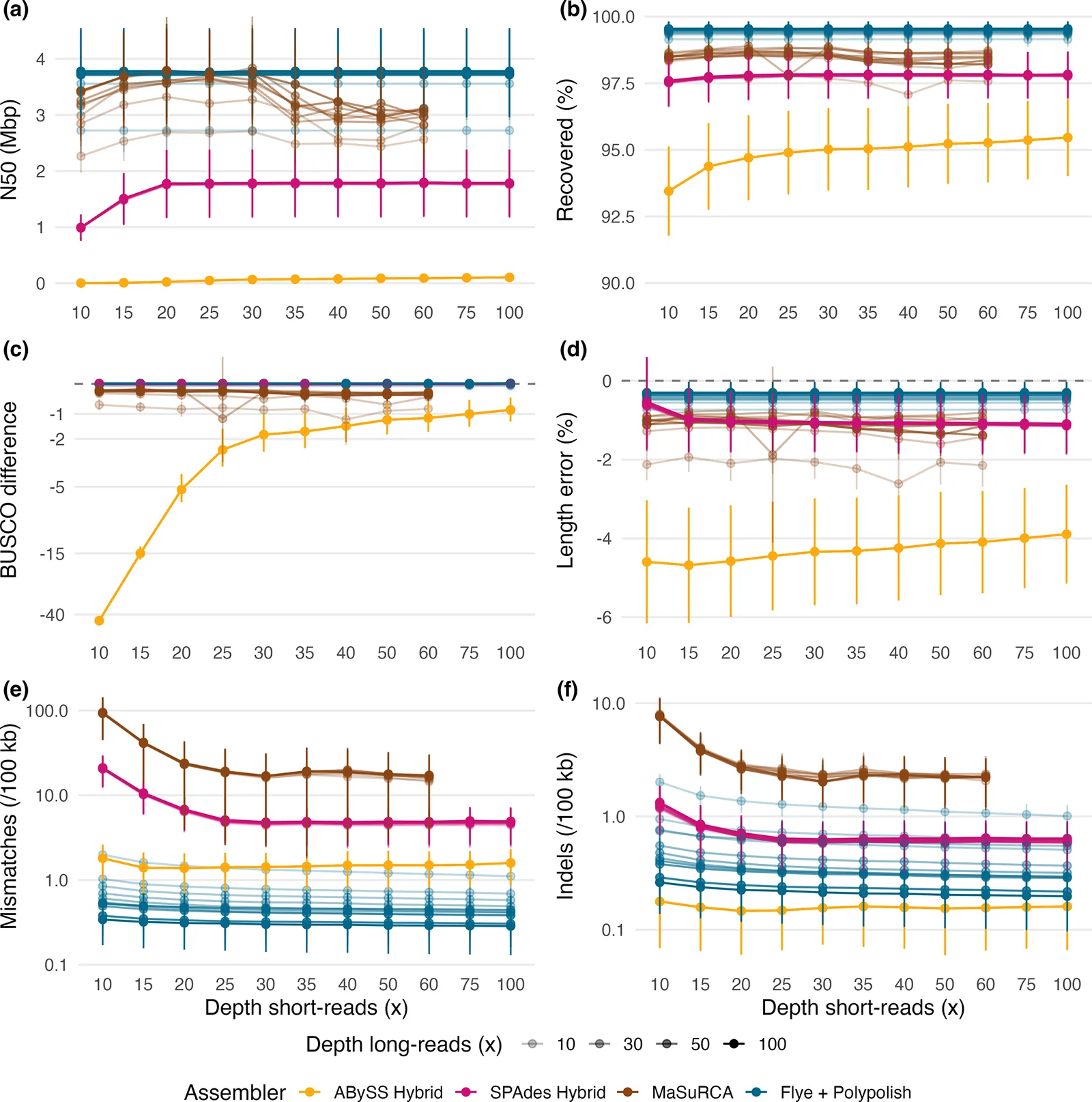
Assembly quality metrics for hybrid assembly strategies. Panels show (**a**) N50, (**b**) genome fraction, (**c**) BUSCO difference, (**d**) length error, (**e**) mismatches per 100 kbp and (**f**) indels per 100 kbp for each assembler as a function of sequencing depth. Error bars represent 95% CIs around the mean, x-axis indicates SR depth and transparency indicates LR depth. Note the y-axis on panels (c), (e) and (f) have been log scaled for better visualization. Hybrid assemblers exhibited clear algorithmic trade-offs: MaSuRCA maximized contiguity, SPAdes hybrid balanced completeness and accuracy and ABySS hybrid minimized error rates but remained fragmented, while Flye+Polypolish provided a high-quality LR benchmark across depths.

Hybrid assembly performance was strongly influenced by algorithm choice. MaSuRCA consistently produced the most contiguous assemblies, reaching 3.53 Mbp (±0.69) Mbp N50 at 30X LR+30 X SR, comparable to polished LR assemblies (Fig. 6a). SPAdes hybrid achieved intermediate contiguity (~1.78 Mbp ±0.60), while ABySS Hybrid remained highly fragmented and never exceeded 0.09 Mbp across depths. Increasing SR depth beyond ~30X did not improve contiguity and in MaSuRCA occasionally reduced it slightly, suggesting diminishing returns or conflict between data types at high SR coverage. These observations were confirmed by the model, which detected significant ‘assembler×LR’ interactions for contiguity (*P*<0.001, Fig. S3C), indicating that hybrid assemblers differ both in baseline scaffolding capability and in their response to LR coverage.

Genome recovery (Fig. 6b) and BUSCO completeness (Fig. 6c) were driven primarily by SR depth and assembler choice. MaSuRCA and SPAdes hybrid recovered ≥97.5% of the genome across depths, whereas ABySS Hybrid lagged slightly (95%). BUSCO differences remained small for SPAdes hybrid (~−0.2 pp) and MaSuRCA (−0.3 pp), indicating near-complete gene recovery. In contrast, ABySS Hybrid showed severe deficits at low coverage (−44.1 pp±1.92 at 10X+10X) but improved with SR depth, reaching −1.15 pp±0.54 at 60X SR. These results indicate that SR support is critical for gene completeness in hybrid assemblies, while LR depth contributes less once sufficient SR coverage is available (*P*<0.001 for assembler and ‘assembler×SR’ effects, Fig. S3C).

**Fig. 7.**
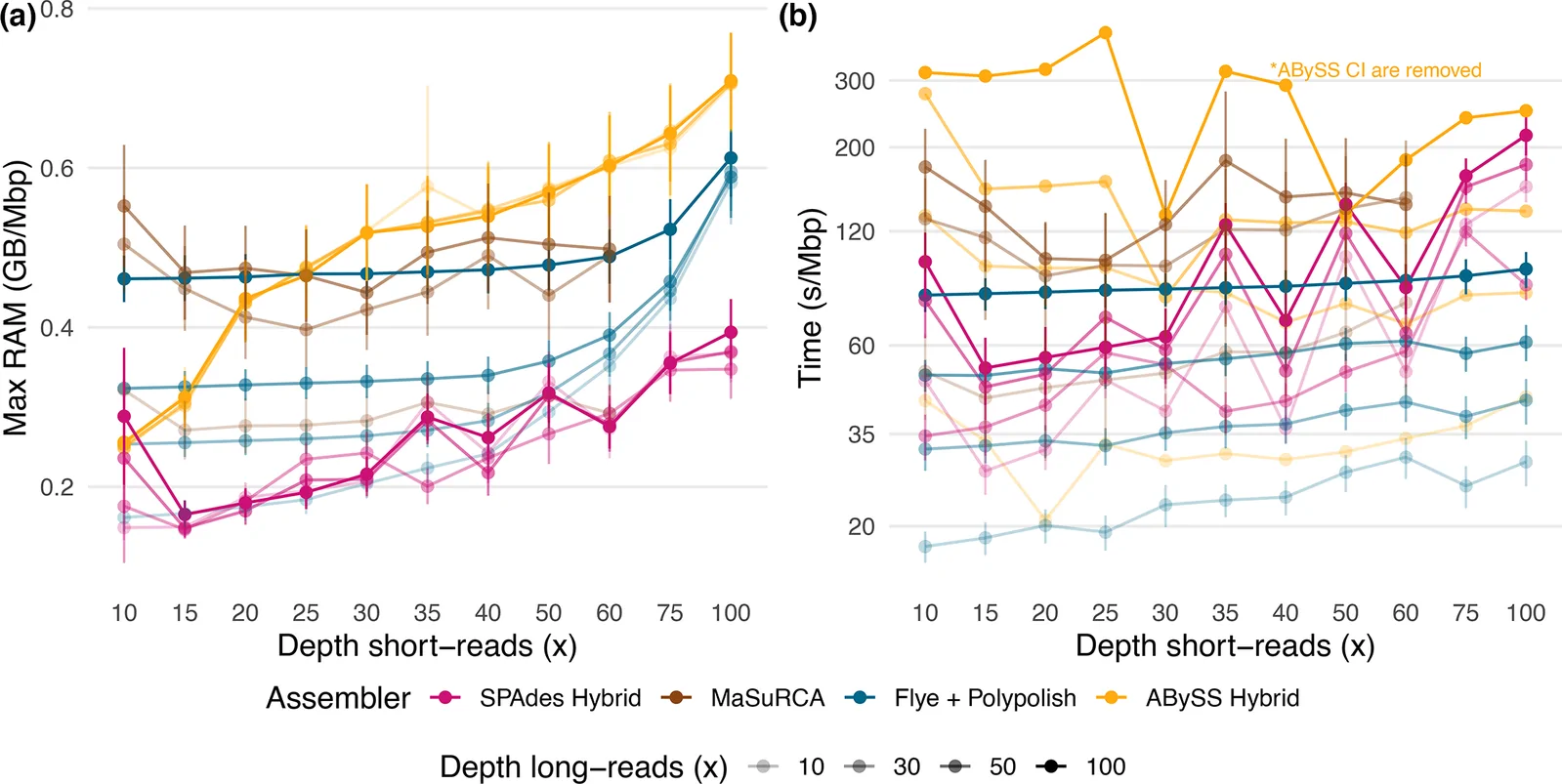
Resource usage per Mbp of assembly for polished and hybrid assembly strategies. Panels show (**a**) run time per Mbp of reference and (**b**) maximum RAM usage per Mbp of reference. Error bars represent 95% CIs around the mean, x-axis indicates SR depth and transparency indicates LR depth. Note the y-axis on panel (b) has been log scaled for better visualization, and CI for ABySS Hybrid removed due to high values. Full depth ranges with all CI are provided in Figs S8 and S9. Computational demand was driven primarily by LR depth and algorithm design: MaSuRCA and ABySS hybrid showed steep runtime and memory scaling at higher LR coverage, whereas SPAdes hybrid and Flye+Polypolish remained comparatively resource-efficient and stable across depths.

Resource usage reflected these algorithmic differences and scaling behaviours (Fig. 7). SPAdes hybrid was the most memory-efficient (~0.15–0.39 GB/Mbp), while ABySS Hybrid required the most RAM at high depths (up to ~0.60 GB/Mbp). MaSuRCA memory usage increased primarily with LR depth but tended to decrease slightly as SR depth increased, suggesting improved read integration efficiency. Runtime scaled steeply with LR depth for MaSuRCA and ABySS (e.g. ABySS reaching 179.9 s/Mbp±192.8 at 60X LR), whereas SPAdes hybrid increased more gradually (~51 s/Mbp±4.6 at 10X LR+60X SR to ~69 s/Mbp±9.1 at 60X LR). These patterns indicate that hybrid assembly computational costs are driven largely by LR data volume and algorithm design rather than SR depth alone (all *P*<0.001, Figs S3C and S7).

Alt text (Fig. 7): Two-panel figure showing normalized computational resource usage per megabase of assembly for polished long-read and hybrid assembly strategies. Panel A displays runtime per mega base pairs, and Panel B shows maximum RAM usage per mega base pairs. Assemblers are compared across sequencing depths, with long-read depth represented in the data points. Resource requirements increase with long-read coverage for some assemblers, particularly MaSuRCA and ABySS hybrid, while SPAdes hybrid and Flye combined with Polypolish show more stable runtime and memory usage across depths.

Overall, hybrid assemblers demonstrate clear algorithmic trade-offs: MaSuRCA maximizes contiguity and completeness, SPAdes hybrid offers balanced performance and efficiency and ABySS Hybrid prioritizes base accuracy but remains fragmented and underestimates genome size. Despite these strengths, none of the hybrid approaches consistently surpassed polished LR assemblies, reinforcing that algorithm selection and workflow design remain the primary determinants of assembly quality.

### Empirical data outcomes

Empirical assemblies reproduced the major depth-dependent patterns observed in simulation, although absolute values showed greater variability due to biological heterogeneity and unknown ground truth. K-mer-based genome size estimates closely matched final assembly lengths across assemblers (Fig. S10), supporting their use as proxies for genome size. SR estimates averaged 36.7 Mb, whereas LR estimates averaged 22.5 Mb, ~38.5% lower. Across assemblies at unsub-sampled depths, final genome lengths typically fell within ~5–15% of the SR estimates, indicating good concordance overall. LR estimates were typically lower than final assemblies, whereas SR estimates more closely matched the assemblies. In several isolates, LR estimates were several megabases smaller than final assemblies, indicating consistent underestimation in those cases. In addition, smaller minimum-overlap parameters in Flye consistently produced longer assemblies, increasing total assembly size by ~15% on average relative to larger overlap settings, although the change in settings affected the isolates differently, indicating that read-length distribution can also affect final assemblies.

**Fig. 8.**
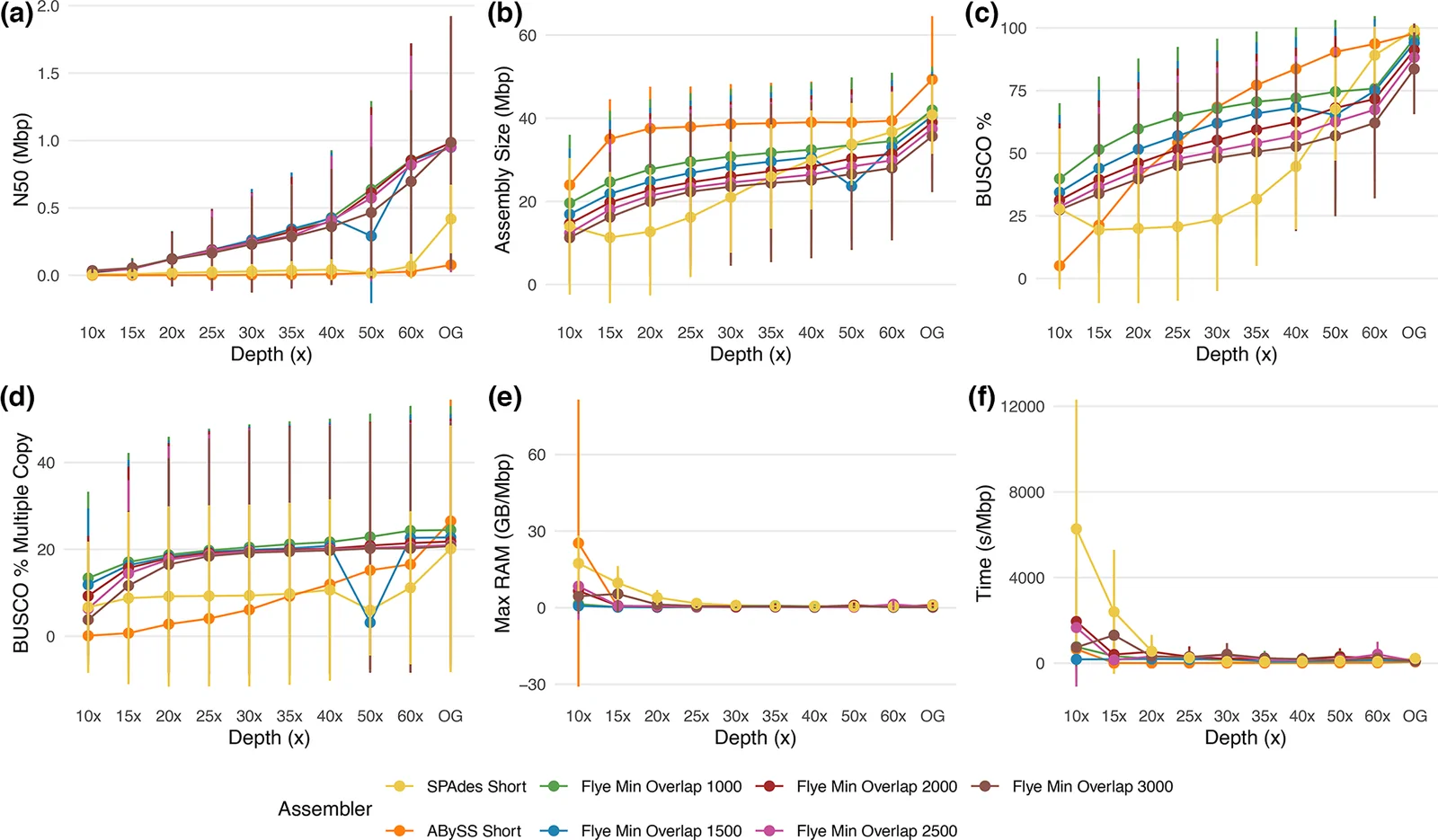
Empirical data single reads strategy summary. Panels show (**a**) N50, (**b**) Assembly Size, (**c**) BUSCO Complete %, (**d**) BUSCO Multi Copy %, (**e**) Max RAM per Mbp and (**f**) Time per Mbp. Error bars represent 95% CIs around the mean. Across empirical isolates, increasing LR depth improved contiguity and completeness while reducing computational cost, with Flye outperforming SR assemblers and minimum-overlap settings influencing assembly size and recovery.

Assembly performance showed significant effects of depth and genome size, as well as ‘assembler×depth’ interactions (all *P*<0.001; Fig. S11), confirming assembler-specific responses to coverage. Contiguity (N50; Fig. 8a) increased with depth for Flye across all minimum-overlap settings and typically plateaued between 20X and 40X coverage, closely matching the plateau range in the simulated data. SR assemblers remained highly fragmented at all depths, reaching similar contiguity only at the unsub-sampled depths (>400X). Assembly size consistently increased with depth (Fig. 8b), with ABySS short producing the longest assemblies but plateauing at a moderate 25X depth. SPAdes short had the lowest sizes until 35X when it reached similar values to Flye with minimum overlap of 3,000 bases. Notably, although SPAdes assembly size was similar to Flye at 35X, they remained highly fragmented, only reaching LR levels at the ultra-high depths of the unsub-sampled assemblies (>400X).

Completeness improved noticeably with depth (Fig. 8c), as depth and genome size significantly influenced BUSCO completeness (*P*<0.001; Figs S11 and S12). Flye assemblies exceeded ~70% BUSCO completeness at moderate coverage (≥25X) under flexible overlap settings, while SR assemblies diverged from one another. ABySS recovered more BUSCO genes than SPAdes and at comparable levels to Flye, while SPAdes required at least 50X to achieve similar results. Furthermore, multi-copy BUSCO percentages remained low overall (Fig. 8d), though variance increased with depth for the LR, while SPAdes and ABySS remained unchanged until the unsubsampled depths, suggesting a stronger but limited duplication artefacts with LR, but strong isolate-specific effects.

Assembly size accuracy and computational demand also stabilized with increasing depth. Unlike the simulated datasets, computational requirements were highest at low coverage and declined rapidly as depth increased, particularly for SPAdes, with both memory (Fig. 8e) and runtime (Fig. 8f) stabilizing beyond ~25–30X. Mixed-effects models confirmed significant assembler, depth and genome size effects on runtime and memory use (*P*<0.05; Figs S11 and S12), indicating that incomplete assemblies at low depth impose greater computational overhead, possibly due to fragmented graph structures and ambiguous paths. Finally, comparison of polished versus unpolished assemblies (Figs S12C–E and S13) showed that polishing primarily improved low-depth assemblies. Base-level corrections were greatest at low LR coverage and increased sharply with initial SR depth before plateauing near ~20–25X, consistent with simulation results.

**Fig. 9.**
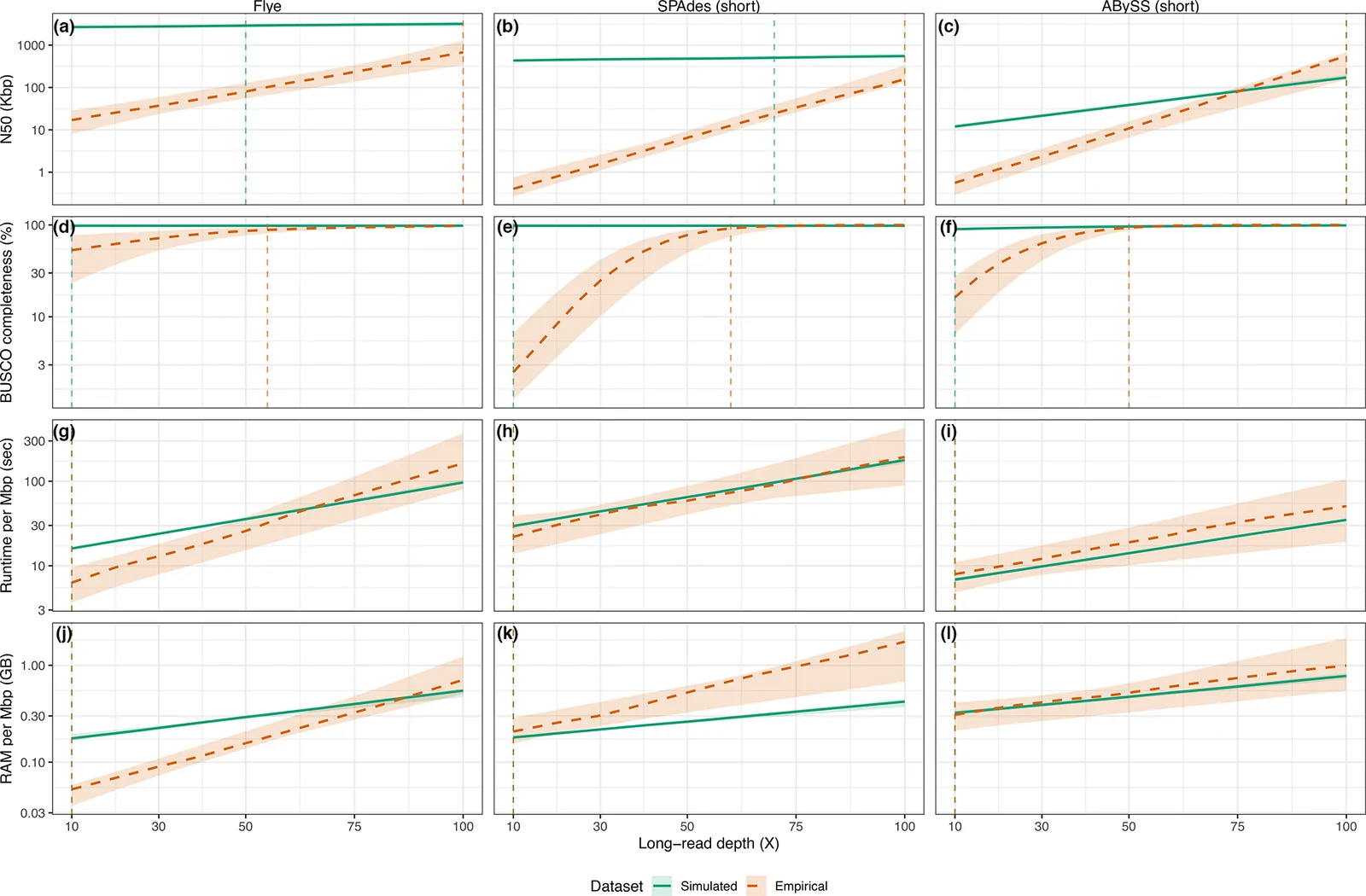
Model-predicted depth-response curves for shared assemblers and metrics across simulated and empirical datasets. Rows represent the assembly metrics: (**a–c**) N50; (**d–f**) BUSCO completeness; (**g–i**) run time; (**j–l**) max RAM. Columns represent different programmes: (**a, d, g, j**) Flye; (**b, e, h, k**) SPAdes short; (**c, f, i, l**) ABySS short. Orange-dashed lines represent the empirical dataset and green-continuous lines represent the simulated dataset. Shaded ribbons represent 95% CIs. Vertical line of each colour represents the depth threshold at which 90% of its best value was achieved. All y-axes use log10 scaling. Simulated predictions for runtime and RAM use reference-normalized metrics, while empirical predictions use proxy-normalized. Predictions were truncated at biological lower bounds where applicable (e.g. non-negative for runtime and RAM).

Across all measured outcomes, empirical assemblies largely followed the qualitative patterns observed in simulation (Fig. 9), confirming that the inferences derived from simulated data translate to field-collected fungal isolates. Most discrepancies were quantitative rather than qualitative: empirical Flye N50 continued to gain through 60X LR coverage where simulated Flye N50 plateaued by 20–30X, and computational requirements at low depth were probably inflated by fragmented graph structures in empirical but not in simulated assemblies. These differences suggest that simulation-derived depth recommendations should be taken as lower bounds for real-world sequencing planning, with empirical fungal assembly likely requiring ~1.5–2X the simulated depth to achieve equivalent contiguity.

## Discussion

This study addresses a gap in genome-assembly by focusing on fungi whose genome architectures (broad size range, variable gene structure and frequent repeats) pose different challenges from the bacterial benchmarks that dominate much of the assembly literature [36–38, 42]. Using a combined framework of simulated and empirical datasets spanning diverse genome architectures, our results showed that assembly outcomes reflect a three-way interplay between sequencing depth, algorithmic design and intrinsic genome properties. In particular, depth effects were strongly assembler-dependent, with LR assemblers showing larger contiguity gains at low-to-moderate LR coverage followed by plateauing, while SR-only assemblers showed comparatively weak responses to increased depth.

These contrasting responses reflect fundamental differences in assembly paradigms: LR assemblers leverage read length to span repeats and resolve structural complexity once sufficient overlap coverage is achieved, while SR assemblers rely on de Bruijn graph connectivity, which remains constrained by repeat length and sequencing errors even at very high coverage. This pattern supports a practical interpretation of fungal assembly performance: additional data does not improve all approaches equally, and sequencing depth must be evaluated alongside assembler error tolerance, repeat-resolution capacity and graph construction strategy.

Across approaches, our results point to diminishing returns in LR depth once moderate coverage is achieved. In both simulated and empirical datasets, most improvements in contiguity and completeness occurred by ~20–40X LR depth (Fig. 9), after which additional data produced only marginal improvements despite increased computational cost. Similar saturation patterns have been reported in LR benchmarking studies, where assembly contiguity and completeness improve rapidly at low-to-moderate coverage but plateau once repeat-spanning coverage and graph connectivity are achieved [37, 38, 75, 76]. Importantly, this ‘coverage-to-benefit’ curve differed among assemblers, reinforcing that ‘optimal depth’ is not a single number but depends on the algorithm’s sensitivity to read errors, repeat structure and coverage heterogeneity.

Furthermore, SR polishing presents an efficient alternative to pushing LR depth beyond this moderate range. In the simulated polishing analyses (Flye baseline), SR depth and polisher choice strongly shaped mismatch/indel outcomes, while contiguity and genome recovery were largely inherited from the underlying LR assembly (Figs 3–5, S3B and S5). Similarly, in the empirical comparisons, polishing base corrections were higher in lower-depth LR assemblies, with sharp increased gains at lower SR depths before plateauing (Fig. S10). These results reinforce prior reports that polishing can deliver large base-level accuracy improvements at relatively modest additional sequencing cost, often approaching maximal benefit at moderate SR depths [32, 77]. Among polishers, Polypolish consistently provided the strongest improvements, while Racon outcomes strongly depended on mapper choice, emphasizing that seemingly ‘upstream’ decisions can propagate to measurable differences in final accuracy, a point often emphasized in workflow comparisons in genomics [78–80].

Hybrid assembly results further illustrate how algorithm choice shapes outcomes even when the data types are fixed. Hybrid assemblers showed greater variability than other assembler types, reflecting differences in how each assembler balances SR and LR information, and resulting in clear trade-offs in contiguity, accuracy and computational cost (Figs 6 and 7). MaSuRCA tended to maximize contiguity, consistent with its strategy of constructing super-reads and then leveraging LRs for scaffolding/bridging [53], but this came with higher error rates and heavier computational demands in our benchmarks. In contrast, hybridSPAdes generally produced less contiguous but cleaner assemblies, consistent with a SR-first-assembly backbone with cautious LR integration to fill gaps and resolve repeats [33], thereby trading contiguity for improved base-level accuracy. By comparison, ABySS hybrid attempts to rescaffold SR draft contigs using LR data [48], resulting in high base-level accuracy but highly fragmented assemblies, suggesting that its strategy may under-utilize LR information relative to other hybrid approaches. Overall, our data suggest that algorithm selection can meaningfully shape assembly structure and error profiles even when sequencing depth and data composition are held constant, reinforcing the importance of evaluating multiple workflows rather than relying on a single default pipeline.

Genome-to-genome variation further indicates that assembly success is not uniform across fungal taxa and that genome characteristics can modulate both assembly outcomes and computational requirements. In the simulated data, genome size and gene density were consistently associated with greater variability in contiguity, completeness and computational demand (Figs 4c, d, 5d, e and 7d, e), indicating that larger and more structurally complex genomes require proportionally greater sequencing and computational resources. These relationships help explain part of the dispersion observed in the empirical datasets and further suggest that there is no single best solution and that recommendations should be scaled with genome complexity. Although our models do not include a dedicated repeat-content term, repeat and transposable-element content is a known driver of assembly-graph ambiguity, particularly for SR approaches [81, 82]. In fungal genomes, these are known to be drivers of genome size and gene density [25, 83, 84], both of which are explicit covariates in our analysis. As such, the genome-architecture effects we show, including the higher computational demand and greater depth requirements for larger genomes, already encompass much of the influence that could be attributed to repeat content. Structurally compact genomes achieved near-maximal performance at lower coverage while larger, intron-rich or repeat-dense genomes benefited more from LR depth and polishing. Despite base composition varying widely across the sampled fungi, its influence on final assembly quality was comparatively limited relative to structural features, suggesting that repeat organization and gene architecture, rather than GC content alone, are the primary determinants of assembly difficulty.

Patterns observed under controlled simulation were largely recapitulated in the empirical datasets for most metrics (Fig. 9), indicating that simulation-based benchmarking can meaningfully anticipate real-world assembly behaviour despite increased biological variability and lack of a known ground truth. The relative performance ranking of major LR assemblers remained consistent across both datasets, and polishing gains plateaued within comparable SR coverage windows (~20 to 25X in simulation vs. ~20X in the empirical data). The clearest departure from this concordance was contiguity, which could be due to the simulated reads being generated from finished reference assemblies and therefore inherit its structure. Therefore, repeat arrays, structural variants and heterozygosity that fragment empirical assemblies would already be collapsed in the reference. Additionally, read simulators reproduce an averaged, largely context-independent error model, whereas empirical data are directly influenced by laboratory and sample conditions which can affect sequencing [85]. Simulated N50 consequently saturates at lower depth, while empirical N50 continues to improve as added coverage and read length span these features. Likewise, BUSCO completeness increased rapidly until moderate depth was achieved, although its stabilization while N50 continued to rise suggests that core genomic content had already been retrieved, and further quality improvements resulted from closing gaps between the contigs.

Although absolute metric values varied among isolates due to genome-specific features and laboratory variability, the agreement in performance trends indicates that controlled simulation can serve as a practical planning heuristic for fungal genome assembly. This concordance aligns with broader benchmarking efforts in genomics that emphasize the utility of simulated datasets for evaluating algorithmic behaviour [39, 42]. Our results extend this conclusion to eukaryotic microbial genomes where structural complexity and repeat content introduce additional assembly challenges.

Overall, our data suggest that laboratories can optimize their sequencing strategies by targeting moderate depths (e.g. 30–40X LR+10–20X SR) to achieve high-quality assemblies with manageable costs and computational burdens. Platform selection, however, is often shaped by local infrastructure, budget and experimental scale. SR technologies remain highly standardized with lower per-base costs and high base-level accuracy, but often depend on centralized instrumentation or external core facilities to realize their throughput advantages [86, 87]. In contrast, LR platforms such as the MinION often require lower initial capital investment and offer greater portability and flexibility for in-house or field-based sequencing, making them attractive options (Table 2). Currently, the newest Oxford Nanopore Technologies MK1D platform is priced ~$5,000 (reagents and flow cells included), while SR’s entry-level iSeq 100 ($20,000) has been phased out, making the MiSeq i100 ($50,000) the cheapest option. Furthermore, this Nanopore option is much more portable, allowing for flexible deployment in both laboratory and field settings [88]. For fungal genomes, which are typically smaller than mammalian or plant genomes, the cost difference becomes less pronounced, although laboratory effort can also differ. SR sequencing tolerates fragmented DNA, simplifying extraction protocols, whereas LR sequencing typically requires high-molecular-weight DNA with higher quality to maximize read length. This makes a significant difference for fungi, whose tough cell walls and presence of secondary metabolites can complicate DNA recovery [89–91]. Although combining SR and LR data consistently produced the most reliable assemblies in this study, dual-platform workflows increase library preparation effort and overall cost and may not be necessary for all projects. Instead, our results indicate that carefully balancing sequencing depth, platform choice and algorithm selection can often achieve comparable assembly quality without excessive investment in raw data volume alone.

**Table 2.** Comparison of practical considerations across sequencing and assembly strategies

Strategy	Device cost	Cost per Gbp	Library cost
Illumina	$50 k (MiSeq i100)	$150–500	$150–200
	$335 k+ (NextSeq 2000)	$25–40	$90–120
Nanopore	$3 k (MK1D)	$15–30	$200–300
	$28 k (PromethION 2 Solo)	$5–15	

Costs are approximate 2025 market estimates based on publicly available provider pricing and typical commercial sequencing service rates, rather than instrument purchase costs alone. Depending on the provider, these costs may include consumables, library preparation, sequencing reagents, instrument operation and service overhead.

## Conclusions

This study demonstrates that, for fungal genomes, sequencing depth alone does not determine assembly quality; algorithm choice and genome architecture exert an equal or greater influence on assembly quality. LR sequencing delivered the biggest gains in contiguity at moderate depths (~20–30X), and adding modest SR-based polishing (~10–20X) was usually sufficient to achieve high base accuracy without substantial increases in cost or computational demand. Assembly performance varied with genome size and gene density, which were associated with differences in both contiguity and computational demands, although GC content played a comparatively minor role relative to structural features. Together, these results indicate that effective assembly design should balance software selection with the biological complexity of the target genome rather than relying on sequencing depth alone. For most fungal genomes, ~30–40X LR depth combined with ~10–20XSR polishing provides an efficient compromise between quality, cost and computational burden. Within this framework, the Flye+Polypolish workflow showed the most consistent performance across simulated and empirical datasets, outperforming alternative polishers and many hybrid approaches overall. Nevertheless, workflow selection can be tuned to match available computational resources, genome complexity and project priorities, and recommendations should remain adaptable as new assemblers and polishing strategies emerge.

## Supplementary material

10.1099/mgen.0.001824 Supplementary Material 1.

10.1099/mgen.0.001824 Supplementary Material 2.

## Abbreviations

CDS: coding sequence
CI: confidence interval
LR: long read
RAM: random access memory
SR: short read
